# Synthesis and Anti-Platelet Activity of Thiosulfonate Derivatives Containing a Quinone Moiety

**DOI:** 10.3797/scipharm.1411-14

**Published:** 2015-02-24

**Authors:** Khrystyna Bolibrukh, Svyatoslav Polovkovych, Omar Khoumeri, Tetyana Halenova, Irina Nikolaeva, Olexiy Savchuk, Thierry Terme, Patrice Vanelle, Vira Lubenets, Volodymyr Novikov

**Affiliations:** 1 Department of Technology of Biologically Active Substances, Pharmacy and Biotechnology, Lviv Polytechnic National University, Bandera Str. 12, 79013, Lviv, Ukraine; 2 Laboratoire de Pharmaco-Chimie Radicalaire, Institut de Chimie Radicalaire ICR, Faculté de Pharmacie, Aix-Marseille Univ, CNRS, UMR 7273, Bd J. Moulin 27, 13385, Marseille Cedex 05, France; 3 Educational and Scientific centre “Institute of Biology”, Taras Shevchenko National University of Kyiv, Volodymyrska Str. 64/13, 01601, Kyiv, Ukraine

**Keywords:** Thiosulfonate, Quinone, Anti-platelet activity

## Abstract

Thiosulfonate derivatives based on quinones were synthesized for studying “structure-activity relationship” compounds with an acylated and a free amino-group. Anti-platelet activity of the synthesized compounds was determined and the influence of substituents on the activity of the derivatives was assessed.

## Introduction

It is widely recognized that platelet activation and aggregation plays a crucial role in the initiation and progression of ischemic diseases [1–3]. Given the increasing morbidity and mortality from acute ischemic syndromes, anti-platelet agents have been extensively researched and developed. There are a large number of drugs that can interfere with platelet function [4–7]. Unfortunately, all the currently available anti-platelet agents have unique adverse effects and less than ideal efficacy. So, additional studies are needed and new selective platelet inhibitors with an increased anti-thrombotic efficiency and safety profile must be developed. It is known that compounds with the thiosulfonate moiety show antithrombotic, antimicrobial, and antitumour properties [8–12]. The natural and synthetic derivatives of quinones play a considerable role, as they are the units of vitamins, antitumor drugs, and also quinones which are anti-platelet agents with different mechanisms of action [13–15].

**Fig. 1:**
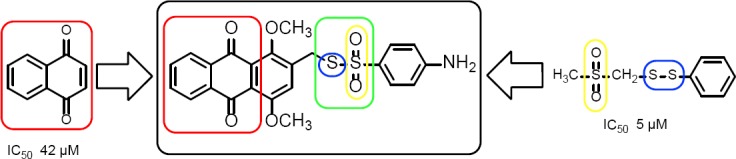
Design of new thiosulfonate derivatives of quinones

In paper [15], it was shown that the wide diapason of antithrombotic activity of quinone derivatives was caused by the nature of the substituent. On the other hand, sulfur-rich compounds that contain the R-SO_2_-CH_2_-S-S-R fragment are also known as the agents that possess antithrombotic behavior [8]. An interesting fact in this case is the decrease in corresponding activity due to the increasing number of methylene groups between the sulfonyl and sulfide fragments. In both research works, compounds with a high index of platelet anti-aggregation were found. Therefore, we decided to synthesize derivatives that contain the quinoid system of bonds and a thiosulfonate fragment in one molecule, and then study their antithrombotic activity. Combining such pharmacophors (Figure 1) is interesting both from synthetic and pharmacological significance points of view.

## Results and Discussion

### Chemistry

Herein we report the synthesis of new thiosulfonate derivatives of quinones by the interaction of the corresponding salts of thiosulfoacids that have properties of S,N-binucleophiles with quinone derivatives. Selected salts of thiosulfoacids, as bifunctional molecules, can react with the halogen derivatives of quinones by two alternative ways: both by the thiosulfonate fragment and also by the amino group. The choice of the solvent and reaction conditions predetermine the direction of the substitution.

In optimizing parameters of the reaction, we based it on our previous works in the synthesis of thiosulfonate derivatives [16–20].

The target of this work was to synthesize derivatives by the S-nucleophilic center. Experimentally, it was found that the highest yields of the S-substituted products using the sodium salt of 4-aminobenzenethiosulfonic acid **2a** were obtained while carrying out the reaction in THF at room temperature. The general scheme of transformations is shown below:

**Sch. 1:**
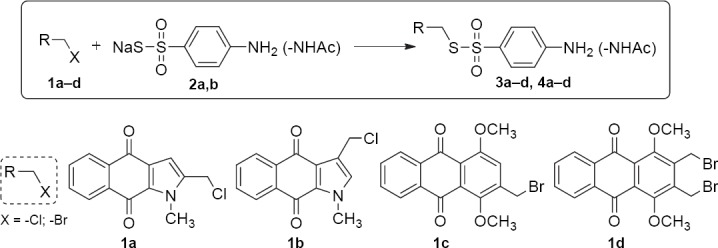
Synthesis of quinoid compounds based on the salt of 4-amino/acetylamino-benzenethiosulfonic acid

The formation of the products was monitored by TLC and LC-MS analysis. The NMR spectrum of quinoid derivatives of thiosulfonates showed signals of the amino group in diapason from 6.09–6.34 ppm.

By studying the influence of an acetyl group on the biological activity, the derivatives **4a–d** were synthesized. For excluding the possibility of the obtained products of N-alkylation, we directly used the sodium salt of 4-(acetylamino)benzenethiosulfonic acid **2b**, which allowed us to carry out the reaction in DMF, and due to its high polarity, caused rapid dissociation of the S-Na bond with the formation of the thiosulfonate anion and -CH_2_-Hal bond with the formation of the corresponding carbocation.

### Biological Activity

Platelet function can be regulated by various agonists. A major signaling molecule causing platelet aggregation is adenosine-5’-diphospate (ADP), which activates platelets and is known to play an important role in hemostasis and thrombosis. Moreover, ADP receptor antagonists are widely in clinical use. That is why the activities of new thiosulfonate derivatives of quinones as inhibitors of ADP-induced platelet aggregation were assayed.

Adenosine diphosphate (ADP), an important platelet agonist *in vivo*, has two types of G protein-coupled receptors in the platelet plasma membrane: P2Y1 and P2Y12 [21]. The end result of ADP signaling through its P2Y1 receptor is an activation of the events that lead to the initiation of platelet aggregation. The P2Y12 receptor appears to play a crucial role in the amplification of platelet aggregation and stabilization of formed aggregates. Moreover, the P2Y12 receptor is responsible for most of the potentiating effects of ADP when platelets are activated by agonists such as collagen and thrombin. From a clinical perspective, P2Y12 is more important, since pharmacological studies demonstrate that this receptor is the molecular target of anti-thrombotic drugs. Several drugs targeting the P2Y12 receptor (e.g. clopidogrel and prasugrel, which are thienopyridines, as well as cangrelor and ticagrelor, which are nonthienopyridines) are now being used in clinical settings given their beneficial effects in patients with atherosclerosis, myocardial infarction, and cerebral vascular diseases [5, 6, 22, 23]. Unfortunately, all the current available ADP receptor antagonists have different side effects such as moderate action, high inter-individual variability in pharmacological response, and severe bleeding risk that limited them only for emergency use. Therefore, there is a requirement for further improvement of anti-platelet treatment and development of novel anti-platelet agents with an increased efficacy and safety profile.

In rabbit platelet-rich plasma (PRP), the maximal changes in light transmission observed at the ADP (5 μmol/L) was 46±4% (control). The data suggested that the vehicle 1% DMSO had no effect on platelet aggregation induced by ADP (Table 1). Our results have shown that, among the series, compound **3c** had the best inhibitory activity against platelet aggregation and the degree of inhibition was proportional to its concentration. Compounds **3d** and **4d** inhibited ADP-induced platelet aggregation only at high concentration (100 μmol/L). In contrast, the closely structurally related analogs, compounds **4b** and **4c**, were not effective in inhibiting platelet aggregation in the same assay at both concentrations (Table 1). These results suggest that the presence of a free amino group in the thiosulfonate moiety of the tested quinones is associated with higher anti-platelet activity. We identified that **3c**, but not other tested derivatives of quinones, may be a new potent anti-platelet agent.

**Tab. 1:**
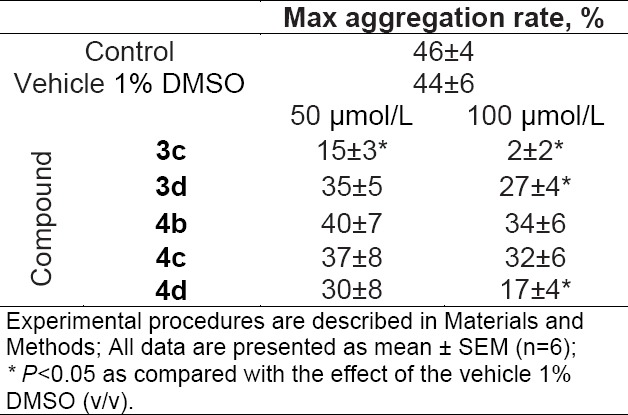
Effect of new thiosulfonate derivatives of quinones on *in vitro* ADP-induced platelet aggregation in rabbit PRP

To investigate the IC_50_ of derivative **3c**, samples of citrated PRP were pre-incubated with vehicle 1% DMSO or with increasing concentrations of the studied compound (final concentration: 5, 10, 25, 30, 50, 75, 100 μM) for 2 minutes. The aggregation was induced by adding ADP (final concentration: 5 μM) and we monitored the change of light transmission for 8 minutes, measuring the maximal increase after the addition of the inducer. The concentration at which the test compound showed 50% inhibition was taken as the IC_50_.

**Fig. 2:**
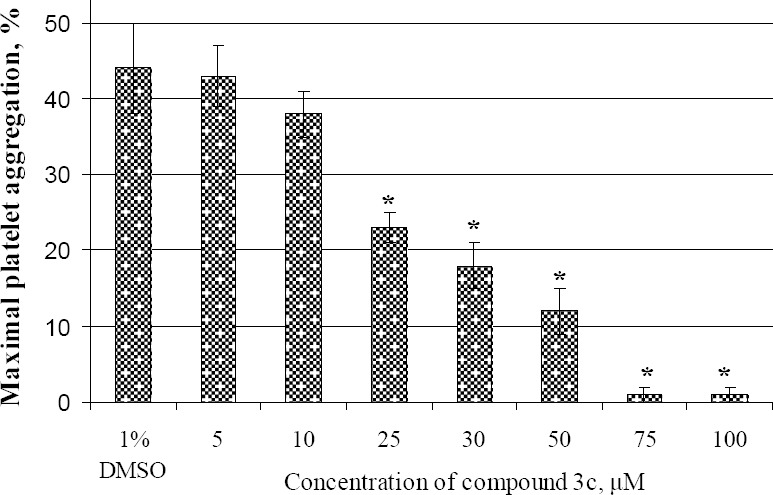
Maximal ratio of ADP-induced platelet aggregation after 2 min incubation of PRP with different concentrations of derivative **3c** or with 1% DMSO. All data are presented as mean ± SEM (n=5). * *P*<0.05 as compared with the effect of DMSO 1% (v/v).

Our results suggest that the degree of inhibition was proportional to the concentration of compound **3c** (Figure 2). As shown in Figure 2, the inhibition increased linearly from 10 to 75 μM with the half maximal inhibitory concentration (IC_50_) – 25 μM.

To study the time-dependent inhibitory effect of derivative **3c** on ADP-induced platelet aggregation, samples of PRP were pre-incubated with 50 μM of the studied compound for 0, 2, 3, 5, 20, 40, and 60 minutes at 37°C with continuous stirring. The aggregation was induced by adding 5 μM ADP and monitored for the maximal changes in light transmission.

**Fig. 3:**
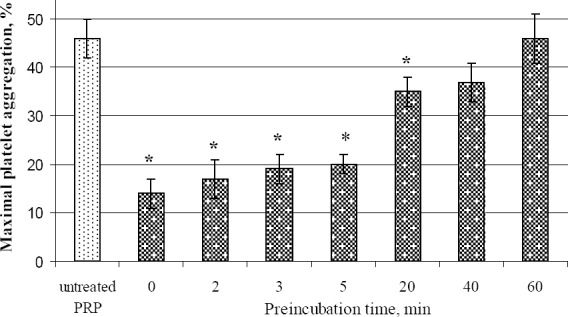
Maximal ratio of ADP-induced platelet aggregation after pre-incubation of PRP with 50 μM of the studied quinoid thiosulfonate derivative for 0, 2, 3, 5, 20, 40, 60 min. All data are presented as mean ± SEM (n=5). * *P*<0.05 as compared with untreated PRP.

As shown in Figure 3, the inhibition levels of ADP-induced aggregation observed after pre-incubation of PRP with 50 μM of compound **3c** for 0, 2, 3, or 5 minutes did not differ from each other. On the other hand, the inhibitory effect of the test compound was significantly reduced after more than twenty minutes of incubation. Moreover, after pre-incubation of PRP with 50 μM of the derivative for 60 minutes, the level of aggregation was identical to that in untreated PRP (Figure 3).

In this report, we describe the synthesis and evaluation of new thiosulfonate derivatives of quinones, that were obtained by the interaction of the sodium salt of 4-amino or 4-(acetylamino)benzenethiosulfonic acid **2a,b** with corresponding methyl halogenated quinones **1a–d**. *In vitro* platelet aggregation experiments were carried out to measure the effects of the new thiosulfonate derivatives of quinones on platelet function. A study of biological properties of these compounds revealed that one member of the group (**3c**) had considerable activity as an inhibitor of ADP-induced platelet aggregation. We have established that this compound had less activity compared with that of the clinically used one. But, unlike the thienopyridine derivatives, which irreversibly bind to P2Y12 receptors, thereby prolonging bleeding time, compound 3c is a reversible ADP receptor antagonist and perhaps may be safer.

The research of the structure-activity relationships of the synthesized compounds allowed the establishment of certain interesting facts about the nature of active groups that provide anti-platelet activity. Herein, it was revealed that the free amino group and -S-SO_2_- fragment predetermined the anti-platelet activity of the investigated products, compared with the results given in [8, 15]. The knowledge about the structure of the active compound will be used for further development of more effective agents in this series. We hope that the new thiosulfonate derivatives of quinones may provide an improved alternative to currently available ADP receptor inhibitors.

## Experimental

### Chemistry

Melting points were determined on a Büchi capillary melting point apparatus and remain uncorrected. Element analyses were performed by the Centre of Microanalyse of the Aix-Marseille University. Both ^1^H- and ^13^C-NMR spectra were determined on a Bruker AC 200 spectrometer. The ^1^H the ^13^C chemical shifts are reported from CDCl_3_ peaks: ^1^H (7.26 ppm) and ^13^C (77.16 ppm) and from DMSO peaks: ^1^H (2.50 ppm) and ^13^C (39.52 ppm).

The following adsorbents were used for column chromatography: silica gel 60 (Merck, particle size 0.063–0.200 mm, 70–230 mesh ASTM). TLC was performed on 5 cm × 10 cm aluminum plates coated with silica gel 60 F254 (Merck) in an appropriate solvent.

### General procedure for the synthesis of 4-aminobenzenethiosulfonic acid S-(R-methyl) esters 3a–d and 4-(acetylamino)benzenethiosulfonic acid S-(R-methyl) esters 4a–d:

Into a two-necked flask equipped with a nitrogen inlet was added solution of the appropriate methyl halogenated derivative **1a–d** in THF (or DMF) and dissolved in a portion of THF (or DMF) sodium salt of 4-aminobenzenethiosulfonic acid **2a** (or 4-(acetylamino)benzenethiosulfonic acid **2b**). The solution was stirred and maintained at room temperature for 5 hours. After this time, TLC analysis showed that compound **2a** (or **2b**) was totally consumed. The reaction mixture was treated with ice water and extracted three times with dichloromethane. The organic phase was washed with water, and then dried over anhydrous sodium sulfate. After evaporation, the product was purified by silica gel chromatography and recrystallized from ethanol and gave corresponding 4-amino-benzenethiosulfonic acid S-(R-methyl) esters **3a–d** (or 4-(acetylamino)benzenethiosulfonic acid S-(R-methyl) esters **4a–d**).

By these procedure compounds **3a–d** and **4a–d** were synthesized:

#### 4-Aminobenzenesulfonothioic acid S-(1-methyl-4,9-dioxo-4,9-dihydro-1H-benzo[f]indol-2-yl-methyl) ester 3a

Eluent: dichloromethane–diethyl ether: 97.5:2.5. Yellow crystals, mp 183–185°C, yield 70%. ^1^H-NMR (200 MHz, DMSO-d6) δ, ppm: 3.89 (s, 3H, N-CH_3_), 4.46 (s, 2H, CH_2_), 6.34 (bs, 2H, NH_2_), 6,52 (s, 1H, Ar-H), 6.58 (d, *J* = 8.6 Hz, 2H, Ar-H), 7.50 (d, *J* = 8.6 Hz, 2H, Ar-H), 7.77–7.81 (m, 2H, Ar-H), 7.99–8.07 (m, 2H, Ar-H). ^13^C-NMR (50 MHz, DMSO-d6) δ: 28.21 (CH_2_), 33.87 (N-CH_3_), 104.02 (CH), 114.31 (2CH), 126.28 (C), 127.63 (CH), 127.89 (CH), 129.80 (2CH), 131.21 (C), 131.93 (C), 133.47 (C), 134.72 (C), 134.90 (CH), 135.21 (CH), 135.74 (C), 152.38 (C), 174.61 (CO), 181.23 (CO). Calculated for (C_20_H_16_N_2_O_4_S_2_), %: C 58.24; H 3.91; N 6.79; O 15.52; S 15.55. Found, %: C 58.37; H 4.02; N 6.73; S 15.72.

#### 4-Aminobenzenesulfonothioic acid S-(1-methyl-4,9-dioxo-4,9-dihydro-1H-benzo[f]indol-3-yl-methyl) ester 3b

Eluent: dichloromethane–diethyl ether: 90:10. Grey-yellow crystals, mp 204–207°C, yield 32%. ^1^H-NMR (200 MHz, DMSO-d6) δ, ppm: 3.85 (s, 3H, N-CH_3_), 4.30 (s, 2H, CH_2_), 6.55 (d, *J* = 8.8 Hz, 2H, Ar-H), 7.00 (s, 1H, Ar-H), 7.44 (d, *J* = 8.8 Hz, 2H, Ar-H), 7.71–7.78 (m, 2H, Ar-H), 7.91–7.97 (m, 2H, Ar-H). ^13^C-NMR (50 MHz, DMSO-d6) δ: 30.49 (CH_2_), 36.42 (CH_3_), 112.51 (2CH), 118.29 (C), 123.83 (C), 126.02 (CH), 126.10 (CH), 129.38 (2CH), 130.21 (C), 132.69 (C), 133.23 (CH), 133.30 (CH), 133.51 (CH), 133.59 (C), 154.22 (C), 175.01 (CO), 180.68 (CO). Calculated for (C_20_H_16_N_2_O_4_S_2_), %: C 58.24; H 3.91; N 6.79; O 15.52; S 15.55. Found, %: C 58.32; H 3.97; N 6.72; S 15.69.

#### 4-Aminobenzenesulfonothioic acid S-[(9,10-dihydro-1,4-dimethoxy-9,10-dioxo-2-anthracenyl)methyl] ester 3c

Eluent: dichloromethane–diethyl ether: 90:10. Yellow crystals, mp 215–217°C, yield 30%. ^1^H-NMR (200 MHz, DMSO-d6) δ, ppm: 3.70 (s, 3H, OCH_3_), 3.83 (s, 3H, OCH_3_), 4.33 (s, 2H, CH_2_), 6.22 (s, 2H, NH_2_), 6.60 (d, *J* = 8.6 Hz, 2H, Ar-H), 7.39 (s, 1H, Ar-H), 7.55 (d, 2H, *J* = 8.6 Hz, Ar-H), 7.79–7.84 (m, 2H, Ar-H), 7.97–8.06 (m, 2H, Ar-H). ^13^C-NMR (50 MHz, DMSO-d6) δ: 34.54 (CH_2_), 56.82 (OCH_3_), 62.71 (OCH_3_), 114.03 (2CH), 120.37 (CH), 125.42 (C), 126.44 (CH), 126.61 (CH), 127.13 (C), 129.59 (CH), 133.02 (C), 133.41 (CH), 133.63 (C), 133.84 (2CH), 134.21 (C), 138.29 (C), 151.10 (C), 152.32 (C), 156.17 (C), 182.73 (CO), 183.03 (CO). Calculated for (C_23_H_19_NO_6_S_2_), %: C 58.84; H 4.08; N 2.98; O 20.45; S 13.66. Found, %: C 58.02; H 4.41; N 2.76; S 12.85.

#### 4-Aminobenzenesulfonothioic acid S,S’-[(9,10-dihydro-1,4-dimethoxy-9,10-dioxo-2,3-anthracenediyl)bis(methylene)] ester 3d

Eluent: dichloromethane-diethyl ether: 85:15. Yellow crystals, mp 121–124°C, yield 40%. ^1^H-NMR (200 MHz, DMSO-d6) δ, ppm: 3.64 (s, 6H, 2OCH_3_), 4.32 (s, 4H, 2CH_2_), 6.12 (s, 4H, 2NH_2_), 6.62–6.69 (m, 4H, Ar-H), 7.72–7.78 (m, 4H, Ar-H), 7.84–7.88 (m, 2H, Ar-H), 8.04–8.07 (m, 2H, Ar-H). ^13^C-NMR (50 MHz, DMSO-d6) δ: 27.31 (2CH_2_), 61.78 (2CH_3_), 113.86 (4CH), 122.83 (2C), 126.71 (2CH), 129.40 (2CH), 134.94 (2CH), 135.62 (2C), 135.91 (2C), 135.83 (2C), 152.61 (2C), 154.83 (2C), 182.30 (2CO). Calculated for (C_30_H_26_N_2_O_8_S_4_), %: C 53.72; H 3.91; N 4.18; O 19.08; S 19.12. Found, %: C 53.57; H 3.84; N 4.24; S 18.89.

#### 4-(Acetylamino)benzenesulfonothioic acid S-[(4,9-dihydro-1-methyl-4,9-dioxo-1H-benz[f]indol-2-yl)methyl] ester 4a

Eluent: dichloromethane–diethyl ether: 95:5. Yellow crystals, mp 208–211°C, yield 55%. ^1^H-NMR (200 MHz, DMSO-d6) δ: 1.82 (s, 3H, CH_3_), 3.87 (s, 3H, N-CH_3_), 4.57 (s, 2H, CH_2_), 6.40 (s, 1H, Ar-H), 7.56 (d, *J* = 8.9 Hz, 2H, Ar-H), 7.67 (d, *J* = 8.9 Hz, 2H, Ar-H), 7.76–7.80 (m, 2H, Ar-H), 7.96–8.02 (m, 2H, Ar-H), 10.13 (s, 1H, NH). ^13^C-NMR (50 MHz, DMSO-d6) δ: 24.09 (CH_3_), 31.03 (CH_2_), 33.10 (N-CH_3_), 109.51 (CH), 118.04 (CH), 118.23 (CH), 126.11 (CH), 126.32 (CH), 126.76 (C), 128.09 (2CH), 130.92 (C), 132.94 (C), 133.62 (2CH), 133.71 (C), 135.70 (C), 137.32 (C), 144.31 (C), 168.93 (C(O)N), 175.04 (CO), 179.70 (CO). Calculated for (C_22_H_18_N_2_O_5_S_2_), %: C 58.14; H 3.99; N 6.16; O 17.60; S 14.11. Found, %: C 58.37; H 3.78; N 6.26; S 14.23.

#### 4-(Acetylamino)benzenesulfonothioic acid S-[(4,9-dihydro-1-methyl-4,9-dioxo-1H-benz[f]indol-3-yl)methyl] ester 4b

Eluent: dichloromethane-diethyl ether: 85:15. Yellow crystals, mp 224–227°C, yield 70%. ^1^H-NMR (200 MHz, DMSO-d6) δ: 2.07 (s, 3H, CH_3_), 3.87 (s, 3H, N- CH_3_), 4.44 (s, 2H, CH_2_), 7.15 (s, 1H, Ar-H), 7.68 (dd, *J* = 4.4 Hz, 4H, Ar-H), 7.78–7.82 (m, 2H, Ar-H), 7.97–8.04 (m, 2H, Ar-H), 10.33 (s, 1H, NH). ^13^C-NMR (50 MHz, DMSO-d6) δ: 24.41 (CH_3_), 31.04 (CH_2_), 36.40 (N-CH_3_), 117.56 (C), 118.32 (2CH), 123.78 (C),126.04 (CH), 126.15 (CH), 128.44 (CH), 130.30 (C), 133.01 (CH), 133.19 (C), 133.42 (C), 133.74 (CH), 133.81 (CH), 137.70 (C), 144.21 (C), 169.30 (C(O)N), 175.12 (CO), 180.74 (CO). Calculated for (C_22_H_18_N_2_O_5_S_2_), %: C 58.14; H 3.99; N 6.16; O 17.60; S 14.11. Found, %: C 58.43; H 3.82; N 6.37; S 14.03.

#### 4-(Acetylamino)benzenesulfonothioic acid S-[(9,10-dihydro-1,4-dimethoxy-9,10-dioxo-2-anthracenyl)methyl] ester 4c

Eluent: dichloromethane–diethyl ether: 80:20. Orange crystals, mp 207–212°C, yield 30%. ^1^H-NMR (200 MHz, DMSO-d6) δ: 1,97 (s, 3H, CH_3_), 3.70 (s, 3H, OCH_3_), 3.82 (s, 3H, OCH_3_), 4.43 (s, 2H, CH_2_), 7.40 (s, 1H, Ar-H), 7.68 (d, *J* = 9.0 Hz, 2H, Ar-H), 7.77–7.88 (m, 4H, Ar-H), 7.98–8.06 (m, 2H, Ar-H), 10,10 (s, 2H, NH). ^13^C-NMR (50 MHz, DMSO-d6) δ: 24.34 (CH_3_), 34.60 (CH_2_), 56.67 (OCH_3_), 62.21 (OCH_3_), 118.53 (2CH), 121.30 (CH), 126.14 (CH), 126.22 (2CH), 126.61 (C), 128.49 (2CH), 133.40 (C), 133.67 (CH), 134.01 (CH), 134.13 (C), 137.32 (C), 137.96 (C), 144.54 (C), 151.69 (C),155.50 (C), 169.22 (C), 181.44 (CO), 182.31 (CO). Calculated for (C_25_H_21_NO_7_S_2_), %: C 58.70; H 4.14; N 2.74; O 21.89; S 12.54. Found, %: C 58.83; H 4.02; N 2.67; S 12.62.

#### 4-(Acetylamino)benzenesulfonothioic acid S,S’-[(9,10-dihydro-1,4-dimethoxy-9,10-dioxo-2,3-anthracenediyl)bis(methylene)] ester 4d

Eluent: dichloromethane-diethyl ether: 80:20. Orange crystals, mp 214–217°C, yield 22%. ^1^H-NMR (200 MHz, DMSO-d6) δ: 2.00 (s, 6H, 2CH_3_), 3.63 (s, 6H, 2OCH_3_), 4.41 (s, 4H, 2CH_2_), 7.72–7.85 (m, 10H, Ar-H), 8.03 (s, 2H, Ar-H), 10.37 (s, 2H, 2NH). ^13^C-NMR (50 MHz, DMSO-d6) δ: 24.63 (2CH_3_), 31.72 (2CH_2_), 63.01 (2OCH_3_), 119.04 (4CH), 126.56 (2CH), 128.68 (4CH), 133.75 (2C), 134.50 (2CH), 136.32 (2C), 137.31 (2C), 144.90 (2C), 155.21 (2C), 169.59 (2CO), 181.72 (2CO). Calculated for (C_34_H_30_N_2_O_10_S_4_), %: C 54.10; H 4.01; N 3.71; O 21.19; S 16.99. Found, %: C 54.19; H 4.23; N 3.64; S 16.87.

### Measurement of Anti-Platelet Activity

#### Preparations of platelets

All procedures were conducted at room temperature. Blood was collected from the auricular artery of healthy rabbits into 3.8% citrate in a ratio of 9:1 and then centrifuged at 150 g for 15 min in order to obtain platelet-rich plasma (PRP). The PRP was carefully removed and placed into a plastic tube. Platelet-poor plasma (PPP) was prepared by further centrifugation of the remaining plasma at 1500 g for 40 minutes. Throughout all the experiments, the platelet number was adjusted to 250 x 10-9/L by diluting PRP with PPP.

#### Measurement of platelet aggregation

Platelet aggregation in PRP was recorded under constant stirring conditions (500 rpm) at 37°C for 10 min by the aggregometer (AT-02, Belarus). The baseline value was set using PRP while PPP served as a full transmittance control. The PRP suspension was incubated with the studied compounds (final concentration: 50 μmol/L and 100 μmol/L) or with dimethylsulphoxide (DMSO) alone for 2 minutes. The aggregation was induced by adding ADP (final concentration: 5 μmol/L) and the change of light transmission was monitored for 8 minutes, measuring the maximal increase after the addition of the inducer. Concentrations of ADP leading to maximal aggregation were determined in preliminary experiments. To minimize the effect of DMSO, the solvent, on the aggregation, the final concentration of DMSO was fixed at 1% (v/v). In each experiment, all the samples were tested in triplicate.

#### Statistical analysis

Results were expressed as the mean±SEM. The difference between groups was analyzed by the standard Student’s t-test. P-values less than 0.05 were considered statistically significant.

## Authors’ Statement

### Competing Interests

The authors declare no conflict of interest.
